# Biotechnology in Environmental Monitoring and Pollution Abatement 2015

**DOI:** 10.1155/2015/963803

**Published:** 2015-10-07

**Authors:** Kannan Pakshirajan, Eldon R. Rene, Aiyagari Ramesh

**Affiliations:** ^1^Department of Biosciences and Bioengineering, Indian Institute of Technology Guwahati, Guwahati, Assam 781039, India; ^2^Department of Environmental Engineering and Water Technology, UNESCO-IHE Institute for Water Education, Westvest 7, 2611 AX Delft, Netherlands

## 1. Introduction

Rapid industrial growth has led to elevated discharges of toxic chemicals and nutrients in water bodies. The level of a particular pollutant discharged into water bodies depends on industrial activities in the vicinity. Industries such as textiles, mining, tanneries, metal plating, fertilizer and agroindustries, batteries, pesticides, ore refineries, petrochemicals, and paper manufacturing are amongst others that contribute greatly to soil, sediment, air, and water pollution problems. Some of the chemicals are not biodegradable and therefore tend to accumulate in tissues and bioaccumulate in the food chain. This results in health problems in human beings and death of aquatic organisms. In water bodies, the presence of nitrogen and phosphorus increases the production of biomass in aquatic systems, thereby impairing the water quality and threatening the natural balance of these ecosystems. Although stringent nitrogen and phosphorus discharge standards from wastewater have been set in many countries, industries often face problems in meeting these requirements. From the regulatory perspective of a particular country, it is necessary to develop new or optimize the existing wastewater treatment technologies for compliance with the latest discharge standards.

The demand for the use of sustainable and ecofriendly environmental processes is rapidly growing subjected to economic, public, and legislation pressure. Biotechnology provides a plethora of opportunities for effectively addressing issues pertaining to the monitoring, assessment, modeling, and treatment of contaminated soil, sediment, air, and water streams. The different biotechnologies available nowadays represent both well-established and novel (bio)technologies, although several aspects of their performance remain to be tested, for instance, the use of novel biocatalysts and reactor designs, a fundamental understanding of microbial community dynamics and mechanisms occurring within a (bio)reactor, the assessment of the performance of (bio)reactors during long-term operation, and its modeling [1–6]. If these mechanisms are understood and the knowledge gap is bridged, novel biotechniques will potentially change the way users rebuild technologies for the sustainable use of different biological processes for soil, sediment, air, and wastewater treatment.

## 2. Industrial Wastewater Treatment

Agricultural runoff, livestock operations, aquaculture, food processing facilities, pulp and paper mills, sewage treatment plants, and fossil fuel combustion are some typical examples of industries that cause nutrient pollution. In agricultural areas, N, P, and K compounds are easily transported by farmland drainage and surface water to valuable water resources resulting in the deterioration of water quality. C/N ratio is an important parameter to design biological wastewater treatment systems, particularly for treating high N containing wastewater. Sometimes, for unbalanced C/N ratios, exogenous carbon sources can be added to the wastewater. Low cost biological matrices such as woodchips, peanut shells, and barley grains can be added as potential carbon donors. With regard to the use of bioreactors for nitrogen removal, sequencing batch reactors (SBRs) can be used to achieve nitrification under aerobic conditions and denitrification under anoxic conditions. In order to accomplish enhanced nitrogen removal in SBRs, activities of both nitrification and denitrification should be studied during long-term system operation.  Table 1 presents the papers accepted under this category and their authors.

## 3. Control and Assessment of Environmental Pollution

It is a well-known fact that sewage water from various sources is discharged into urban rivers or streams. Conventionally, the water quality index approach is one of the best tools to determine the water quality of the water bodies. Recent studies have offered ingenious and innovative solution for the rehabilitation of urban water bodies and to improve their quality. Bacterial technology (BT) can rehabilitate urbanized water bodies such as lakes, rivers, and streams. BT offers the following advantages; (i) sustainable and reliable for public health, (ii) low maintenance, (iii) minimal operational costs, and (iv) reproducible on any scale of the operation. Titles of papers accepted under this category along with the authors are represented in Table 2.

Concerning the presence of phenolic compounds in soils, they are the most abundant plant metabolites and are believed to decompose slowly in soils compared to other soil organic matters. One of the papers selected for this special issue reviews the turnover rate of phenolics and its quantification in soils, together with their regulatory effects on decomposition. The authors reviewed the following aspects: (i) various structures and forms of phenolics in soils; (ii) extraction and analysis of phenolics in soil samples; (iii) phenolics biodegradation; (iv) effect of phenolics on soil organic matter decomposition; (v) effect of environmental changes, such as elevated CO_2_, global warming, N deposition, and drought, on phenolics decomposition; and (vi) suggestions for future phenolics studies.

The guest editors firmly believe that the collection of papers presented in this special issue will stimulate interest amongst the global research community and would help peers in their research pursuits. Besides, there is an urgent need to translate most of the laboratory-based research into field-based research in order to witness sustainable solutions to persisting environmental problems. Future research should address crucial issues pertaining to (i) nanobiomatrices for environmental remediation, (ii) development of biosensors for environmental monitoring, (iii) development of new biocatalysts (bacteria, fungi, and algae) for environmental applications, (iv) clean practices and development of technologies for pollution prevention, and (v) studies on life cycle assessment (LCA), risk assessment, health, and safety impact assessment.

## Figures and Tables

**Table 1:** 

Title of the accepted manuscript	Authors
“Characteristics of Biological Nitrogen Removal in a Multiple Anoxic and Aerobic Biological Nutrient Removal Process”	H. Wang et al.
“Evaluation of Natural Materials as Exogenous Carbon Sources for Biological Treatment of Low Carbon-to-Nitrogen Wastewater”	J. Ramírez-Godínez et al.
“Decolorization of Distillery Spent Wash Using Biopolymer Synthesized by *Pseudomonas aeruginosa* Isolated from Tannery Effluent”	C. David et al.
“Enhancing Ecoefficiency in Shrimp Farming through Interconnected Ponds”	R. H. Barraza-Guardado et al.

**Table 2:** 

Title of the accepted manuscript	Authors
“Integrated Evaluation of Urban Water Bodies for Pollution Abatement Based on Fuzzy Multicriteria Decision Approach”	S. Hashim et al.
“Vulnerability Assessment and Application of Bacterial Technology on Urban Rivers for Pollution Eradication”	S. Hashim et al.
“The Regulation by Phenolic Compounds of Soil Organic Matter Dynamics under a Changing Environment”	K. Min et al.
